# Interval post-colonoscopy colorectal cancer following a negative colonoscopy in a fecal immunochemical test-based screening program

**DOI:** 10.1055/a-2136-6564

**Published:** 2023-10-04

**Authors:** Hilliene J. van de Schootbrugge-Vandermeer, Arthur I. Kooyker, Pieter H. A. Wisse, Iris D. Nagtegaal, Hiltje A. Geuzinge, Esther Toes-Zoutendijk, Lucie de Jonge, Emilie C. H. Breekveldt, Anneke J. van Vuuren, Folkert J. van Kemenade, Christian R. B. Ramakers, Evelien Dekker, Iris Lansdorp-Vogelaar, Manon C. W. Spaander, Monique E. van Leerdam

**Affiliations:** 1 Department of Public Health, Erasmus MC University Medical Center, Rotterdam, The Netherlands; 2 Department of Gastroenterology and Hepatology, Erasmus MC University Medical Center, Rotterdam, The Netherlands; 3 Department of Pathology, Radboud University Medical Centre, Nijmegen, The Netherlands; 4 Department of Gastrointestinal Oncology, Netherlands Cancer Institute, Amsterdam, The Netherlands; 5 Department of Pathology, Erasmus MC University Medical Center, Rotterdam, The Netherlands; 6 Department of Clinical Chemistry, Erasmus MC University Medical Center, Rotterdam, The Netherlands; 7 Department of Gastroenterology and Hepatology, Amsterdam University Medical Centers, location AMC, Amsterdam, The Netherlands; 8 Department of Gastroenterology and Hepatology, Leiden University Medical Center, Leiden, The Netherlands

## Abstract

**Background** In the Dutch colorectal (CRC) screening program, fecal immunochemical test (FIT)-positive individuals are referred for colonoscopy. If no relevant findings are detected at colonoscopy, individuals are reinvited for FIT screening after 10 years. We aimed to assess CRC risk after a negative colonoscopy in FIT-positive individuals.

**Methods** In this cross-sectional cohort study, data were extracted from the Dutch national screening information system. Participants with a positive FIT followed by a negative colonoscopy between 2014 and 2018 were included. A negative colonoscopy was defined as a colonoscopy during which no more than one nonvillous, nonproximal adenoma < 10 mm or serrated polyp < 10 mm was found. The main outcome was interval post-colonoscopy CRC (iPCCRC) risk. iPCCRC risk was reviewed against the risk of interval CRC after a negative FIT (FIT IC) with a 2-year screening interval.

**Results** 35 052 FIT-positive participants had a negative colonoscopy and 24 iPCCRCs were diagnosed, resulting in an iPCCRC risk of 6.85 (95 %CI 4.60–10.19) per 10 000 individuals after a median follow-up of 1.4 years. After 2.5 years of follow-up, age-adjusted iPCCRC risk was approximately equal to FIT IC risk at 2 years.

**Conclusion** Risk of iPCCRC within a FIT-based CRC screening program was low during the first years after colonos-copy but, after 2.5 years, was the same as the risk in FIT-negative individuals at 2 years, when they are reinvited for screening. Colonoscopy quality may therefore require further improvement and FIT screening interval may need to be reduced after negative colonoscopy.

## Graphical abstract

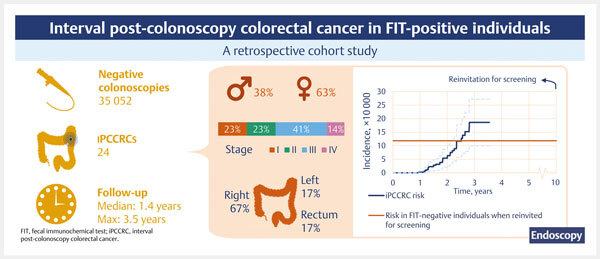

## Introduction

With over 1.9 million cases and 935 000 deaths in 2020, colorectal cancer (CRC) globally ranks third in terms of cancer incidence and second in terms of cancer mortality 1 . To effectively reduce CRC incidence and mortality, population-based screening programs have been launched in many countries 2 3 4 5 6 .

In the Netherlands, individuals aged 55–75 years are biennially invited for fecal immunochemical test (FIT)-based CRC screening. After a positive FIT, participants are referred for a colonoscopy. Based on the findings at colonoscopy, the intensity of the subsequent surveillance strategy is determined. If the colonoscopy is negative, meaning no more than one small adenoma is found, participants are reinvited for FIT-based screening after 10 years. This 10-year screening interval after a negative colonoscopy is in accordance with European guidelines 7 . Moreover, several studies have shown a significantly reduced CRC risk for 10 years or longer after a negative colonoscopy in individuals with low-to-moderate risk 8 9 10 11 12 13 . However, although colonoscopy is the reference standard for the detection of (precursors of) CRC, interval post-colonoscopy CRCs (iPCCRC) may develop 14 15 . Most of these iPCCRCs can be explained by procedural factors, in particular missed lesions at index colonoscopy 16 . As FIT-positive individuals represent a population with a higher a priori risk for advanced adenomas and/or CRC 17 , the risk of iPCCRC is also higher in this population if the colonoscopy miss rate is the same.

Knowledge of the iPCCRC risk in FIT-positive individuals is scarce. Most studies that evaluated iPCCRC risk focused on primary colonoscopy screening 12 14 18 . Few studies examined iPCCRCs in a FIT-based screening program 19 20 . To our knowledge, only one study calculated iPCCRC risk in FIT-positive individuals explicitly, but data were limited and only three iPCCRCs were taken into account 20 . To our knowledge, the risk of iPCCRC in a FIT-based CRC screening program over time has not yet been evaluated.

The fact that FIT-positive individuals have an elevated risk of CRC, leading to a higher risk of iPCCRC in the context of imperfect colonoscopy, raises the question of whether the recommended screening interval of 10 years for individuals at low-to-moderate risk is appropriate for individuals with a negative colonoscopy after a positive FIT. Previous research has suggested that subsequent FIT screening should occur 2 years after negative colonoscopy in FIT-positive individuals if the main purpose is to detect advanced adenomas and/or CRCs missed at colonoscopy 17 . To further investigate this, knowledge about the risk of iPCCRC after a negative colonoscopy in a FIT-based screening program is crucial. Moreover, the CRC risk at which FIT-positive individuals with a negative colonoscopy are reinvited for screening should ideally be in proportion to the CRC risk at which individuals with a negative FIT are reinvited for screening. Therefore, the aim of this study was to assess iPCCRC risk in FIT-positive individuals with a negative colonoscopy in an ongoing screening program. Furthermore, we considered the iPCCRC risk in relation to the risk of interval CRC after negative FIT (FIT IC).

## Methods

### Setting

In the Netherlands, individuals aged 55–75 years have been invited for biennial FIT screening since 2014, according to a phased implementation schedule by birth cohort. The design of the Dutch CRC screening program has been described in detail elsewhere 21 . In brief, after a FIT result above the positivity cutoff of 47 micrograms of hemoglobin per gram feces (µg/g), participants are referred for a colonoscopy. Adherence to colonoscopy following a positive FIT is approximately 82.2 % 22 . Based on findings at colonoscopy and available pathology reports, an adenoma score is assigned to each participant. Participants with an adenoma score of zero, meaning that no relevant findings were detected (see definition below), are reinvited for FIT screening after 10 years 23 . If a colonoscopy is incomplete, a repeat colonoscopy or computed tomography-colonography is performed. Follow-up policies after a colonoscopy with a higher adenoma score include referral for colonoscopy surveillance, or in cases of CRC, referral for cancer treatment.

### Data collection

Screening outcomes, sex, and age of participants who underwent a colonoscopy following a positive FIT between 1 January 2014 and 9 April 2017 were extracted from the Dutch national screening information system (ScreenIT). Only participants with colonoscopies that had an adenoma score of zero and/or with the advice to be reinvited for FIT screening after 10 years were selected. To obtain additional information on findings at colonoscopy, pathology reports of lesions detected at the first and possible repeat colonoscopies were obtained from the Dutch nationwide pathology databank (PALGA) 24 . Through linkage with the Netherlands Cancer Registry, data on all diagnosed CRCs, including primary tumor location and cancer stage, since 1 January 2014 were obtained retrospectively; data up to 6 October 2017 were included.

For individuals who were diagnosed with CRC, we checked whether they already had a CRC diagnosis prior to their FIT invitation; if so, we excluded them from the study as they should not have participated in the screening program.

### Definitions

A negative colonoscopy was defined as a colonoscopy with an adenoma score of zero (i. e. without relevant findings). No relevant findings at colonoscopy indicated the presence of no more than one nonvillous (< 75 % villous component), nonproximal adenoma < 10 mm or serrated polyp < 10 mm. Serrated polyps included hyperplastic polyps, sessile serrated polyps/adenomas, and traditional serrated adenomas. The colon was divided anatomically according to the International Classification of Disease for Oncology (C18–20). Left-sided lesions included lesions located from the rectosigmoid to the descending colon (18.6–7; C19). Proximal lesions were defined as right-sided lesions, and included lesions located from the splenic flexure to the cecum (C18.0; C18.2–5).

An iPCCRC was defined according to the definition of the World Endoscopy Organization consensus as CRC diagnosed at least 180 days after a negative colonoscopy and before the next FIT screening invitation date 25 . In practice, invitation for FIT screening was not yet reached in our data and individuals were followed up until October 2017. CRCs were staged according to the American Joint Committee on Cancer TNM Classification (7th edition until 2017, 8th edition from 2017 onwards) 26 .

### Inclusion process

Negative colonoscopies were characterized by two properties: an adenoma score of zero and referral back to the screening program with reinvitation for FIT screening after 10 years. To correct for incorrect registration of either the adenoma score or the recommendation for referral back to the screening program, we selected study participants with at least one of these two properties. Subsequently, we manually checked all individuals with iPCCRC and a random selection of individuals without iPCCRC to see whether colonoscopies indeed met our definition of a negative colonoscopy based on the pathology reports. Only negative colonoscopies (i. e. colonoscopies with an adenoma score of zero) were included.

**Fig. 1:**
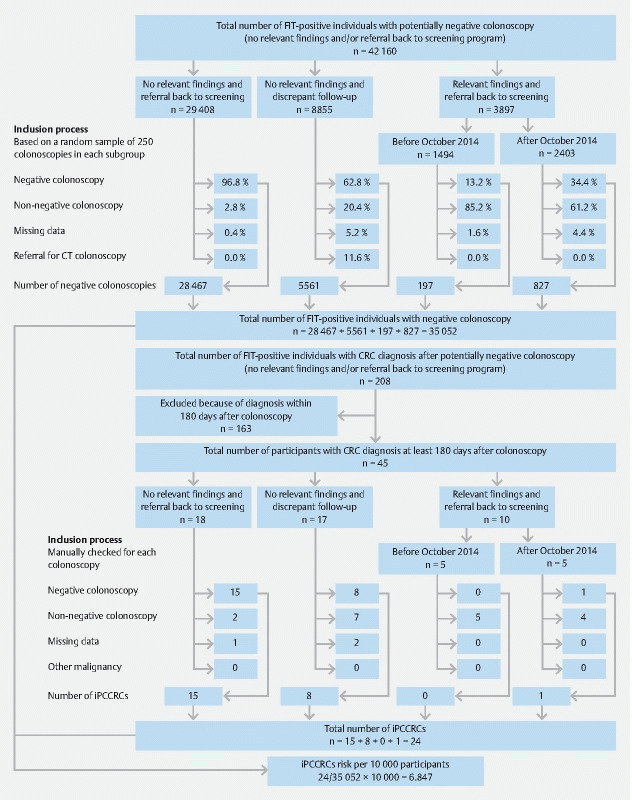
Flow chart describing the manual validation of negative follow-up colonoscopies and interval post-colonoscopy colorectal cancer. FIT, fecal immunochemical test; CRC, colorectal cancer; iPCCRC, interval post-colonoscopy colorectal cancer; CT computed tomography.

Individuals were stratified into subgroups based on their registered colonoscopy outcome and follow-up policy, under the assumption that the rate of negative colonoscopies differed per group ( Fig. 1 ): 1) a group of participants without relevant findings (adenoma score zero) at colonoscopy and with the advice to return to the screening program after 10 years, 2) a group without relevant findings (adenoma score zero) but with discrepant follow-up advice, and 3) a group with relevant findings at colonoscopy but with the advice to return to the screening program after 10 years. The second and third group contained discrepancies between the registered findings at colonoscopy and follow-up policy, which could be a result of incorrect registration by the endoscopist of either colonoscopy findings or follow-up advice. For example, the adenoma score is usually registered by the endoscopist in ScreenIT after receiving the results of the pathology report. However, once the registration in ScreenIT is completed, it cannot be overruled. So, when the endoscopist finds one small distal adenoma and registers an adenoma score of zero, but the pathologist finds > 75 % villous component, the registered adenoma score of zero is incorrect as it should have been higher than zero. The third group was divided further into two groups, based on the colonoscopy date: before or after October 2014. This distinction was made because, at the start of the screening program, endoscopists were technically unable to reject surveillance in ScreenIT when relevant findings were detected at colonoscopy, which could be desirable for example in cases of comorbidity. To not invite those participants for surveillance, the option of referral back to the screening program after 10 years was misused. As a result, it was likely that a large number of participants with relevant findings at colonoscopy, but with the advice to return to the screening program after 10 years, received incorrect follow-up advice and contained few actual negative colonoscopies. After October 2014, the option became available in ScreenIT to indicate no surveillance even if participants had relevant findings. For each subgroup we manually checked a random sample of 250 participants without iPCCRC to see whether the colonoscopies met our definition of a negative colonoscopy, to determine the rate of misclassification in each subgroup. We assumed these samples to be representative for the entire subgroup and used the rates of misclassification to estimate the total number of negative colonoscopies.

All individuals who had an iPCCRC were included in the analysis, even if they would not be reinvited for FIT screening after 10 years due to being older than 75 years, which is the maximum age in the screening program.

### Analyses

We calculated CRC risks per 10 000 participants and per 10 000 person-years of follow-up. iPCCRC risk per participant was calculated as the number of iPCCRCs divided by the total number of negative colonoscopies in all subgroups. Person-years of follow-up after a negative colonoscopy were calculated as the time between colonoscopy and CRC diagnosis or maximum follow-up (i. e. 6 October 2017), and corrected using the rate of misclassified colonoscopies in the corresponding subgroup. iPCCRC risk per person-year of follow-up was calculated as the number of iPCCRCs divided by the sum of corrected person-years of follow-up in all subgroups.

Data from a previous study were used to calculate FIT IC risk, which included age, sex, and FIT result of all participants with negative FIT at a cutoff of 47 µg/g in the first screening round in 2014 27 . To further ensure the reliability of these data, we excluded participants whose FIT analysis date was more than half a year after the invitation date, leading to a slightly smaller population of FIT-negative individuals in our study compared with the original study. Differences in age of participants with iPCCRC vs. FIT IC were tested using a Mann–Whitney *U* test, and other characteristics of (participants with) iPCCRC vs. FIT IC were tested using Pearson’s chi-squared test at a significance level of 5 %. FIT IC risk per participant was calculated as the number of interval CRCs divided by the total number of first-round negative FITs. Person-years of follow-up after a negative FIT were calculated as the time between FIT analysis and CRC incidence or the invitation for a next FIT screening. FIT IC risk per person-year of follow-up was calculated as the number of interval CRCs divided by the total number of person-years of follow-up.

We calculated 95 %CIs using Wilson’s method for binomial proportions for the CRC risks per 10 000 participants and profile likelihood for the CRC risks per 10 000 person-years of follow-up. Moreover, we compared iPCCRC risk with FIT IC risk per 10 000 person-years using a two-proportions *Z* test at a significance level of 5 %. To analyze the pattern of CRC risk over time, we plotted age-adjusted cumulative iPCCRC incidence and FIT IC incidence using the complement of a Kaplan–Meier survival function. All calculated *P* values were two sided.

In a post hoc analysis, we examined the completeness of the index colonoscopies of iPCCRCs using a dataset that contained cecal intubation and bowel preparation of colonoscopies in FIT-positive individuals between 2014 and 2016. These data were retrieved from ScreenIT in the context of another study, which is why the data cover a slightly different period than our original data. Cecal intubation was defined as the photographic documentation of at least two of the three landmarks: appendiceal orifice, ileocecal valve, and terminal ileum. Bowel preparation was considered adequate for a Boston Bowel Preparation Scale score of 6 or higher.

## Results

### Study populations

In total, 42 160 colonoscopies for participants with a positive FIT in the first or second screening round were selected, of which 35 052 colonoscopies met the inclusion criteria ( Fig. 1 ). Males comprised 51.4 % of the selected participants and median age at time of colonoscopy was 67 years (interquartile range [IQR] 63–70 years). A total of 208 CRCs were diagnosed after a negative colonoscopy following a positive FIT, of which 163 were excluded because they were diagnosed within 180 days after colonoscopy. Of the remaining 45 CRCs, 21 (46.7 %) were misclassified and had an adenoma score > 0, whereas 24 (53.3 %) met the definition to qualify as iPCCRC ( Fig. 1 , **Table 1 s** in the online-only Supplementary material).

Furthermore, 370 593 participants with a negative FIT in the first screening round were selected. Males comprised 48.0 % of the selected participants and the median age at time of FIT screening was 65 years (IQR 63–67 years). Within 2 years after negative FIT, 418 interval CRCs were diagnosed ( Table 1 ).

**Table 1:** Characteristics of (participants with) interval post-colonoscopy colorectal cancer and interval colorectal cancer after negative fecal immunochemical test.

	iPCCRC	FIT IC 2	*P* value
Total, n	24	418	
Sex, n (%)			0.23
Male	9 (38) 1	209 (50)	
Female	15 (63)	209 (50)	
Age category, n (%)			< 0.01
55–59 years	0 (0)	0 (0)	
60–64 years	1 (4)	101 (24)	
65–69 years	8 (33)	185 (44)	
70–76 years	15 (63)	132 (32)	
Stage, n (%)			0.59
I	5 (23)	67 (19)	
II	5 (23)	64 (18)	
III	9 (41)	130 (37)	
IV	3 (14)	90 (26)	
Unknown	2 (NA) 3	67 (NA) 3	
Location, n (%)			0.27
Right side	16 (67)	201 (51) 1	
Left side	4 (17)	78 (20)	
Rectum	4 (17)	118 (30)	
Unknown	NA (NA) 3	21 (NA) 3	
Time to diagnosis, days			–
Median (IQR)	700 (480–853)	727 (710–730)	
Time to diagnosis, n (%)			–
180–359 days	1 (4)	–	
360–539 days	6 (25)	–	
540–720 days	5 (21)	–	
> 720 days	12 (50)	–	

iPCCRC, interval post-colonoscopy colorectal cancer; FIT IC, interval colorectal cancer after negative fecal immunochemical test; NA, not applicable; IQR, interquartile range.

1 Percentages do not total 100 % due to rounding.

2 Data on FIT ICs come from Toes et al. (2020) 27 .

3 Not included in the percentage distribution of stage and location

### iPCCRC and FIT IC characteristics

Most iPCCRCs were diagnosed in women (63 %), within age category 70–76 years (63 %), at an advanced stage (55 % in stage III or IV), and located in the right side of the colon (67 %) ( Table 1 ). iPCCRCs were diagnosed at a median of 700 days (IQR 480–853 days) after colonoscopy.

Half of the FIT ICs were found in women (50 %) and one third of the FIT ICs were found within age category 70–76 years (32 %) ( Table 1 ). The age distribution of participants with iPCCRC was significantly different from the age distribution of participants diagnosed with FIT IC.

### iPCCRC and FIT IC risk

iPCCRC risk was 6.85 (95 %CI 4.60–10.19) per 10 000 FIT-positive individuals with a negative colonoscopy after a median follow-up period of 1.4 years (IQR 0.9–2.2 years), and 3.61 (95 %CI 2.35–5.26) per 10 000 person-years of follow-up. iPCCRC risk did not significantly differ between males and females, but increased with age: iPCCRC risk per 10 000 person-years was 0.56, 2.90, and 7.97 in individuals aged 60–64, 65–69, and 70–76 years, respectively ( Table 2 ). FIT IC risk was 11.28 (95 %CI 10.25–12.41) per 10 000 individuals after a median follow-up period of 2.0 years (IQR 1.9–2.0 years), and 5.75 (95 %CI 5.22–6.32) per 10 000 person years of follow-up ( Table 2 ).

**Table 2:** Risk of interval colorectal cancer after negative colonoscopy or negative fecal immunochemical test.

	**iPCCRC**		**FIT IC**		*P* value 1
	N per 10 000 participants (95 %CI)	N per 10 000 person-years of follow-up (95 %CI)	N per 10 000 participants (95 %CI)	N per 10 000 person-years of follow-up (95 %CI)	
All	6.85 (4.60–10.19)	3.61 (2.35–5.26)	11.28 (10.25–12.41)	5.75 (5.22–6.32)	0.03
Sex					
Male	5.06 (2.66–9.62)	2.68 (1.29–4.83)	11.76 (10.27–13.46)	6.00 (5.22–6.85)	0.02
Female	8.68 (5.26–14.32)	4.57 (2.63–7.29)	10.84 (9.46–12.41)	5.52 (4.80–6.30)	0.56
Age category					
55–59 years	–	–	–	–	–
60–64 years	0.97 (0.17–5.49)	0.56 (0.03–2.46)	8.42 (6.93–10.22)	4.32 (3.53–5.21)	0.03
65–69 years	5.53 (2.80–10.91)	2.90 (1.32–5.39)	9.97 (8.64–11.52)	5.10 (4.40–5.87)	0.15
70–76 years	18.07 (10.95–29.79)	7.97 (4.59–12.71)	20.28 (17.10–24.04)	10.16 (8.52–11.99)	0.44

iPCCRC, interval post-colonoscopy colorectal cancer; FIT IC, interval colorectal cancer after negative fecal immunochemical test.

1 Two-proportions *Z* test, where N per 10 000 person years of follow-up is compared between iPCCRC and FIT IC.

**Fig. 2:**
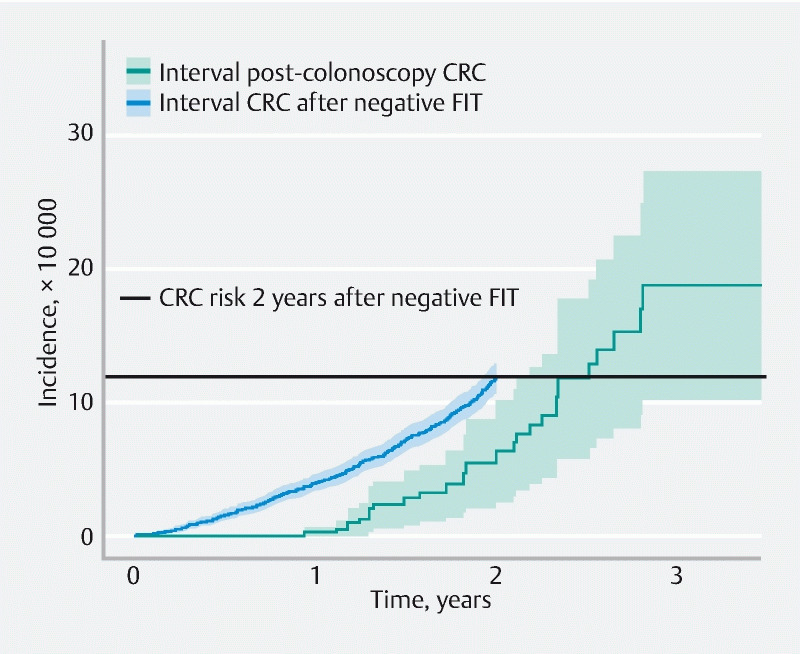
Age-adjusted cumulative colorectal cancer incidence per person-year of follow-up. CRC, colorectal cancer; FIT, fecal immunochemical test.

Interval CRC risk per 10 000 person-years was significantly lower after a negative colonoscopy than after a negative FIT ( Table 2 ). However, after 2.5 years of follow-up, age-adjusted iPCCRC risk was approximately equal to the FIT IC risk in the first screening round at 2 years ( Fig. 2 ).

### Completeness of colonoscopies

In the post hoc analysis, completeness of the index colonoscopies in all 24 participants with iPCCRCs was evaluated. A total of 23 colonoscopies (95.8 %) had cecal intubation and 22 colonoscopies (91.7 %) had adequate bowel preparation ( **Table 1 s** ).

## Discussion

In this study, we assessed iPCCRC risk in FIT-positive individuals in the first years after a negative colonoscopy in an ongoing screening program. We observed an iPCCRC risk of 3.61 per 10 000 person-years of follow-up with a median follow-up period of 1.4 years. The majority of iPCCRCs were diagnosed in women, at an older age, at an advanced stage, and located in the right side of the colon. iPCCRC risk was significantly lower than FIT IC risk (3.61 vs. 5.75 per 10 000 person-years of follow-up). However, the estimated patterns of cumulative interval CRC incidence over time showed that iPCCRC risk after approximately 2.5 years of follow-up was similar to the FIT IC risk at 2 years. For most of the negative colonoscopies after which the iPCCRCs occurred, the cecum was reached (95.8 %) and bowel preparation was adequate (91.7 %).

Literature on iPCCRC risk in FIT-positive individuals is scarce. To our knowledge, there is only one study that explicitly investigated the risk of CRC after a negative colonoscopy following a positive FIT 19 . The authors found an iPCCRC risk of 8 per 10 000 person-years of follow-up, which is higher than our estimation. However, their conclusions were based on a relatively small sample size (three out of 740 FIT-positive individuals with negative colonoscopy developed iPCCRC during a median follow-up period of 4.7 years). Another study implicitly reported on iPCCRCs in FIT-positive individuals while focusing on colonoscopy quality within a FIT-based screening program 28 . iPCCRC risk could be calculated from the data and was approximately 7.41 per 10 000 participants (13/17 540) during a 2-year screening interval after negative colonoscopy. This risk is in line with our estimated risk of iPCCRC, although the exact period of follow-up is unknown and may have been slightly different from that in our study.

As expected, iPCCRC risk in a primary colonoscopy setting appears to be lower than in our study. A Polish study found 1.98 CRCs per 10 000 person-years of follow-up between 0.5 and 5 years after primary colonoscopy screening without neoplastic findings 12 . Similarly, 1.64 CRCs per 10 000 person years of follow-up were found in the United States in the second year after primary colonoscopy screening 29 . This confirms that a positive FIT preselects individuals with a higher risk of CRC. However, comparing the risk of iPCCRC across different studies is challenging owing to the variety of definitions that are used to describe iPCCRC risk 25 30 31 .

Although our data show that iPCCRC risk is low in absolute terms after a median follow-up period of 1.4 years, it rapidly increases during the first years after colonoscopy and, after only 2.5 years, reaches the same level of interval CRC risk found in FIT-negative individuals at the time when they are reinvited for screening (2 years). As maximum follow-up in our data was < 4 years and by design there were no advanced adenomas detected at index colonoscopy, following the World Endoscopy Consensus Statement, the most plausible explanation for the incidence of iPCCRCs in our data is that (precursors of) these cancers were missed at colonoscopy 25 . Therefore, colonoscopy quality is an important factor to consider in the evaluation of iPCCRC incidence. This is underlined by previous Dutch studies, which show that adenoma detection rates and proximal serrated polyp detection rates of endoscopists are inversely related to iPCCRC incidence 32 33 . The importance of colonoscopy quality is also confirmed by the overrepresentation of right-sided colon cancers among iPCCRCs in our data. Explanations for missed right-sided lesions include incompleteness of colonoscopy 14 and the fact that sessile serrated lesions are more frequently located in the right side of the colon. Sessile serrated lesions are often only slightly elevated and have indistinct borders, making them difficult to recognize 34 35 36 .

In addition to drawing attention to colonoscopy quality, our results call for a re-evaluation of the screening interval after negative follow-up colonoscopy. The 10-year interval is currently based on primary colonoscopy screening data. FIT-based screening programs worldwide use various strategies after a negative colonoscopy. However, evidence for the optimal strategy is lacking. Given the higher CRC risk of FIT-positive individuals and the < 100 % accuracy of colonoscopy, there is an urgency for further research to evaluate the optimal FIT-screening interval after a negative colonoscopy in FIT-positive individuals. Several factors should be considered, such as systematic false-positive FIT results, costs, and the capacity of FIT laboratories and colonoscopy centers. Moreover, if iPCCRC risk is to be considered in relation to FIT IC risk, the chosen FIT positivity cutoff should be taken into account, as it is a determinant of FIT IC risk 37 .

This study is the first to evaluate iPCCRC incidence in FIT-positive individuals over time. Strengths of the study include data collection from an organized, population-based CRC screening program, and the large sample size. Additionally, we manually checked whether colonoscopies met our definition of a negative colonoscopy, which increases the reliability of our results.

Nonetheless, this study has several limitations. First, a limited follow-up period was available, as we only had data from 2014 to 2017. Our findings therefore provide preliminary insight into the development of iPCCRC risk in FIT-positive individuals during the first years after colonoscopy, and already indicate that the iPCCRC risk is higher than expected even during a short follow-up period. Second, the data used did not contain detailed information on the quality of the index colonoscopy. As different quality indicators are related to the occurrence of iPCCRCs, we recommend a root-cause analysis of iPCCRCs in the Dutch CRC screening program. In general, all colonoscopies in the Dutch CRC screening program are performed by certified endoscopists in accredited endoscopy centers 38 . Additionally, endoscopy centers are audited yearly to assess compliance with quality requirements 39 . The audit criteria regarding completeness of colonoscopies include that ≥ 95 % of the colonoscopies should have complete cecum intubation and ≥ 90 % of the colonoscopies should have adequate bowel preparation. In a post hoc analysis of the index colonoscopies related to the 24 iPCCRCs, we showed that 95.8 % had complete cecum intubation and 91.7 % had adequate bowel preparation. As these rates are within the audit criteria, we have no reason to assume that the colonoscopies after which the iPCCRCs occurred were of lower quality than other colonoscopies within the Dutch CRC screening program. This should be investigated in more depth in the future, as per World Endoscopy Organization recommendations 25 .

To conclude, we demonstrated that, at a median of 1.4 years of follow-up, iPCCRC risk in FIT-positive individuals is lower than FIT IC risk. However, 2.5 years after negative colonoscopy, the iPCCRC risk reaches the level of FIT IC risk at 2 years. Despite the high quality standards within the Dutch CRC screening program and the yearly audits, (precursors of) the iPCCRCs were most likely missed at index colonoscopy. Our results therefore call attention to the variation in the quality of colonoscopies. As has been suggested, continuous benchmarking of colonoscopy quality standards on an endoscopist level, such as cecal intubation rate and adenoma detection rate, is essential to improve colonoscopy quality in a FIT-based CRC screening setting 33 . Nonetheless, as long as the accuracy of colonoscopy is < 100 %, our results raise the question of whether a shorter screening interval after a negative colonoscopy in a FIT-based CRC screening program would be more appropriate to mitigate the effect of variation in the quality of colonoscopy. A prospective study is needed to weigh the harms and benefits of different FIT-screening intervals in this setting. In the meantime, characteristics of iPCCRCs and corresponding index colonoscopies should be monitored and evaluated in order to continuously improve colonoscopy quality and prevent the occurrence of iPCCRCs.

## Tables 1 s

Supplementary material Zusatzmaterial
