# Supplementary material for: Interval post-colonoscopy colorectal cancer following a negative colonoscopy in a fecal immunochemical test-based screening program
Source: Endoscopy. 2023 Oct 4;55(12):1061–9. doi: 10.1055/a-2136-6564 (PMC10684335; doi:10.1055/a-2136-6564)
Supplement: Supplementary file 1 — Supplementary material [file 22404supmat_10-1055-a-2136-6564.pdf]

Supplementary material

Authors' names *H.J. van de Schootbrugge-Vandermeer, A.I. Kooyker, P.H.A. Wisse, I.D. Nagtegaal, H.A. Geuzinge, E. Toes-Zoutendijk, L. de Jonge, E.C.H. Breekveldt, A.J. van Vuuren, F.J. van Kemenade, C.R.B. Ramakers, Prof. E. Dekker, Lansdorp-Vogelaar, M.C.W. Spaander, M.E. van Leerdam*

Title of paper *Interval post-colonoscopy colorectal cancer following a negative colonoscopy in a fecal immunochemical test-based screening program*

Table 1s Detailed information about participants diagnosed with interval post-colonoscopy colorectal cancer

Patient	Gender	Age	Colonoscopy year	Colonoscopy findings	Adenoma score	Follow-up policy	Days to diagnosis	Location	TNM	Stage	Completeness of colonoscopy
1	Female	71	2016	No abnormalities	0	Back to screening	342	Rectum	Unknown	Unknown	Complete
2	Female	75	2016	No abnormalities	0	Back to screening	408	Right	T3 N1 M1	4	Complete
3	Female	67	2015	Other finding *	0	Back to screening	431	Right	T4 Nx M1	4	Complete
4	Male	70	2016	No abnormalities	0	Back to screening	456	Rectum	T3 N0 Mx	2	Complete
5	Male	65	2014	Serrated polyp	0	Back to screening	473	Rectum	T3 N1 Mx	3	Inadequate bowel preparation
6	Male	70	2016	Non-advanced adenoma	0	Different follow-up	473	Right	T2 N0 Mx	1	Complete
7	Female	75	2014	Hyperplastic polyp	0	Different follow-up	482	Right	T4 N1 Mx	3	Complete
8	Female	63	2015	No abnormalities	0	Back to screening	544	Right	T3 N0 Mx	2	Complete
9	Female	75	2016	No abnormalities	0	Back to screening	576	Right	T3 N1 Mx	3	No cecal intubation
10	Female	67	2014	No abnormalities	0	Back to screening	629	Left	T3 N2 Mx	3	Complete
11	Male	67	2015	Non-advanced adenoma	0	Different follow-up	665	Right	T2 N0 Mx	1	Complete
12	Female	69	2015	Other finding *	0	Back to screening	669	Right	T2 N0 Mx	1	Complete
13	Female	67	2014	Hyperplastic polyp	0	Different follow-up	730	Right	T4 N2 Mx	3	Complete
14	Female	75	2014	Other finding *	0	Different follow-up	766	Right	T3 N2 Mx	3	Complete
15	Male	75	2014	Other finding *	0	Different follow-up	770	Left	Unknown	Unknown	Complete
16	Female	75	2014	Serrated polyp	0	Back to screening	797	Right	T3 N1 Mx	3	Complete
17	Male	76	2014	No abnormalities	0	Back to screening	822	Right	T4 N2 M1	4	Complete
18	Male	67	2014	Other finding *	0	Back to screening	853	Rectum	T2 N0 Mx	1	Complete
19	Female	65	2015	No abnormalities	0	Back to screening	854	Right	T3 N0 Mx	2	Complete
20	Female	75	2014	No abnormalities	0	Back to screening	917	Right	T3 N0 Mx	2	Complete
21	Male	76	2014	Other finding *	1 **	Back to screening	933	Left	T3 N1 Mx	3	Complete
22	Female	74	2014	No abnormalities	0	Back to screening	967	Right	T2 N0 Mx	1	Complete
23	Female	75	2014	Serrated polyp	0	Different follow-up	1022	Right	T3 N0 Mx	2	Inadequate bowel preparation
24	Male	75	2014	Other finding *	0	Different follow-up	1025	Left	T3 N1 Mx	3	Complete

* 1 case of lymphoma and 4 cases with other findings including hemorrhoids, inflammatory polyps or diverticulosis

** Incorrect registration of the colonoscopy in ScreenIT: the pathology report showed that the patient only had an inflammatory polyp without dysplasia
